# A highly attenuated recombinant human respiratory syncytial virus lacking the G protein induces long-lasting protection in cotton rats

**DOI:** 10.1186/1743-422X-7-114

**Published:** 2010-06-02

**Authors:** Myra N Widjojoatmodjo, Jolande Boes, Marleen van Bers, Yvonne van Remmerden, Paul JM Roholl, Willem Luytjes

**Affiliations:** 1 Laboratory of Vaccine Research, Netherlands Vaccine Institute, Bilthoven, The Netherlands; 2 Microscope Consultancy, Weesp, The Netherlands

## Abstract

**Background:**

Respiratory syncytial virus (RSV) is a primary cause of serious lower respiratory tract illness for which there is still no safe and effective vaccine available. Using reverse genetics, recombinant (r)RSV and an rRSV lacking the G gene (ΔG) were constructed based on a clinical RSV isolate (strain 98-25147-X).

**Results:**

Growth of both recombinant viruses was equivalent to that of wild type virus in Vero cells, but was reduced in human epithelial cells like Hep-2. Replication in cotton rat lungs could not be detected for ΔG, while rRSV was 100-fold attenuated compared to wild type virus. Upon single dose intranasal administration in cotton rats, both recombinant viruses developed high levels of neutralizing antibodies and conferred comparable long-lasting protection against RSV challenge; protection against replication in the lungs lasted at least 147 days and protection against pulmonary inflammation lasted at least 75 days.

**Conclusion:**

Collectively, the data indicate that a single dose immunization with the highly attenuated ΔG as well as the attenuated rRSV conferred long term protection in the cotton rat against subsequent RSV challenge, without inducing vaccine enhanced pathology. Since ΔG is not likely to revert to a less attenuated phenotype, we plan to evaluate this deletion mutant further and to investigate its potential as a vaccine candidate against RSV infection.

## Background

Respiratory syncytial virus (RSV) is a non-segmented, negative-stranded RNA virus and a member of the *Paramyxoviridae *family. RSV is the most important cause of serious respiratory tract disease for young infants and children, but also for the elderly and immunocompromised persons. More than 50% of the children are infected within their first year of life. The clinical manifestations range from mild, common cold-like symptoms to more severe bronchiolitis and pneumonia. Clinical observations indicate that the first infection with RSV is generally the most severe, whereas subsequent infections tend to be increasingly milder [1]. The peak incidence of serious disease is at 2 - 6 months of age. RSV infection accounts for 40 - 45% of children hospitalized for bronchiolitis or lower respiratory tract disease [2]. The high disease burden indicates an urgent need for a vaccine against RSV, however there is currently no licensed vaccine available. One major obstacle to the vaccine development is the legacy of vaccine-enhanced disease in a clinical trial in the 1960s with a formalin-inactivated (FI) RSV vaccine. FI-RSV vaccinated children were not protected against natural infection and infected children experienced more severe illness than non-vaccinated children, including two deaths. Studies analyzing the RSV-specific immune response in mice indicate that both Th1 and Th2 CD4 T cell responses, as well as CD8 T cell responses can contribute to RSV vaccine-enhanced disease [3]. Lack of protection by FI-RSV is due to low and poorly neutralizing antibody responses and recently it was shown that low antibody avidity for protective epitopes was caused by poor Toll-like receptor stimulation [4].

Since the trial with the FI-RSV vaccine, various approaches to generate an RSV vaccine have been pursued without success. Attempts include classical live attenuated cold passaged or temperature sensitive mutant strains of RSV, (chimeric) protein subunit vaccines, peptide vaccines and RSV proteins expressed from recombinant viral vectors. Although some of these vaccines showed promising pre-clinical data, no vaccine has been licensed for human use due to safety concerns or lack of efficacy [5,6].

Enhancement of RSV disease does not occur after natural RSV infection and has not been observed after inoculation with live attenuated vaccine candidates [7]. These are important facts in favor of a live attenuated RSV vaccine administered intranasally. The most challenging aspect of developing a live attenuated RSV vaccine is, however, to achieve an appropriate balance between attenuation and immunogenicity in the immunologically immature young infant who possesses varied levels of maternally acquired serum antibodies. Reverse genetics technology holds great promises for the development of such a live attenuated vaccine through its potential of including rationally designed, predetermined changes for vaccine candidates [8].

RSV expresses two major glycoproteins on its surface: the fusion (F) glycoprotein and the attachment glycoprotein (G). RSV F mediates penetration and syncytia formation, while RSV G is the putative attachment protein and is naturally expressed as a membrane-anchored and a secreted form [9]. The F protein is indispensable for virus replication and growth, whereas the G protein is not essential for virus growth *in vitro *[10]. RSV exists as two antigenic subgroups, A and B, with the greatest divergence occurring in RSV G (55% identity between the two RSV antigenic subgroups) [11]. While both F and G are important targets for antibody responses, F is considered to be the major stimulus for virus neutralizing antibodies.

A cold-passaged attenuated RSV subgroup B mutant (*cp52*) was found to replicate well *in vitro *despite the absence of functional G and SH proteins [12]. This vaccine candidate did neither replicate in chimpanzees nor induce neutralizing antibody response in human volunteers after intranasal administration, and was considered to be over-attenuated as a vaccine candidate. In the bovine model, however, a recombinant bovine RSV deletion mutant exclusively lacking the G gene was shown to protect calves against challenge virus replication [13]. A recombinant human RSV lacking the G gene has only been tested in the mouse model [14]. Thus, so far no immunization and challenge studies have been performed with RSV ΔG mutants in a more permissive animal model, such as the cotton rat (*Sigmodon hispidus*). This model has the advantage that RSV replicates well and vaccine-associated enhancement of disease has been well studied [15,16].

Here we describe the construction of a recombinant human RSV (rRSV) and a deletion mutant lacking the G gene (ΔG), based on a recent RSV clinical isolate. These recombinant viruses were compared to wild type virus for replication *in vitro *and their efficacy as live-attenuated vaccine candidates was assessed in the cotton rat model.

## Results

### Generation of rRSV-X and ΔG

**Figure 1:**
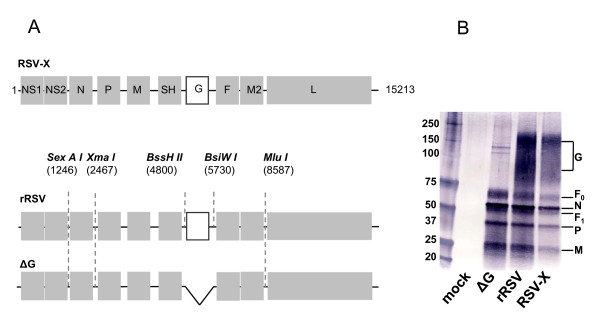
**Generation of recombinant RSV-X virus**. A) Schematic diagram of the RSV-X genome (genome length 15213 nt) and positions of the genetic tags inserted in the cDNA copy of the rRSV-X and ΔG constructs. The *SexA I, Xma I, BssH II, Bsiw I*, and *Mlu I *sites were introduced to facilitate construction. ΔG was recovered by excision of the fragment *BssH II *and *BsiW I *and subsequent religation of the vector. B) Expression of RSV proteins by rRSV and ΔG deletion recombinant viruses. Vero cells were infected at an m.o.i of 0.1 TCID_50_/ml. At 72 hr post infection cell monolayers was harvested and subjected to Western blotting using antiserum against RSV. The molecular weight size markers are depicted on the left and the position of the major RSV proteins are indicated at the right [29].

Clinical isolate 98-25147-X (RSV-X), a RSV serogroup A strain, was used as the basis to create recombinant RSV by reverse genetics. Briefly, the RSV genome of RSV-X was PCR amplified into six large fragments, followed by sequential ligation (Figure 1A). A cDNA recombinant RSV mutant lacking the G gene was subsequently constructed by deletion of the G gene, including its gene-end and gene-start, from the full-length plasmid. To recover recombinant virus plasmids containing the full-length clones were transfected with helper plasmids driving the expression of RSV-A2 N, P, L and M2.1 proteins into MVA-T7 infected Hep-2 cells. To recover and amplify rescued virus, culture supernatants of the transfected Hep-2 cells were used to infect fresh Vero cells. Recovery of the rescued viruses was indicated by immunostaining of infected cells using a polyclonal antibody against RSV (data not shown). The rescued viruses were designated rRSV for the parental recombinant RSV and ΔG for the recombinant RSV mutant lacking the G gene. The identities of the recombinant viruses were verified by sequencing their viral RNA.

Expression of RSV proteins was verified by infecting Vero cells with wild type (wt) RSV-X, rRSV and ΔG followed by Western blotting of cell lysates (Figure 1B). A polyclonal antibody against RSV detected the major RSV structural proteins F, N, P and M in (r) RSV and ΔG infected cell lysates. In addition, as expected, the G protein was present in wt and rRSV, but not in ΔG infected cell lysates. Similarly, cells infected with ΔG did not show expression of the RSV G protein when immunostained with anti RSV-G antibodies (data not shown).

### Replication of recombinant viruses in cell lines

**Figure 2:**
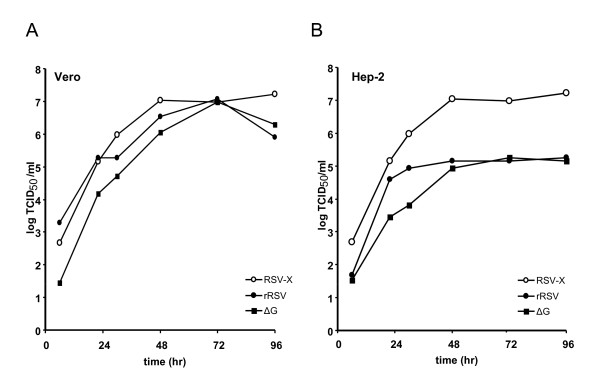
**Growth of (recombinant) RSV in Vero and Hep-2 cells**. Vero (A) and Hep-2 (B) cell monolayers were infected with wild type (wt) RSV, rRSV or ΔG with an MOI of 0.1 and incubated at 37°C. Cells were harvested at the indicated time points and virus TCID_50 _titers were determined in Vero cells.

Replication of the recombinant viruses was characterized on Vero and Hep-2 cells (Figure 2). In Vero cells, growth kinetics of rRSV and ΔG were similar to that of the wild type virus. Peak titers of 10^7 ^TCID_50_.ml^-1 ^were reached 72 hr post infection. Both recombinant viruses were attenuated on Hep-2 cells, as shown by a lower growth rate and 100-fold lower peak titers compared to the wild type virus. The ΔG virus did not form distinct syncytica, infection in Vero cells resulted in areas of rounded cells similarly as described by Teng *et. al. *[10](data not shown).

**Figure 3:**
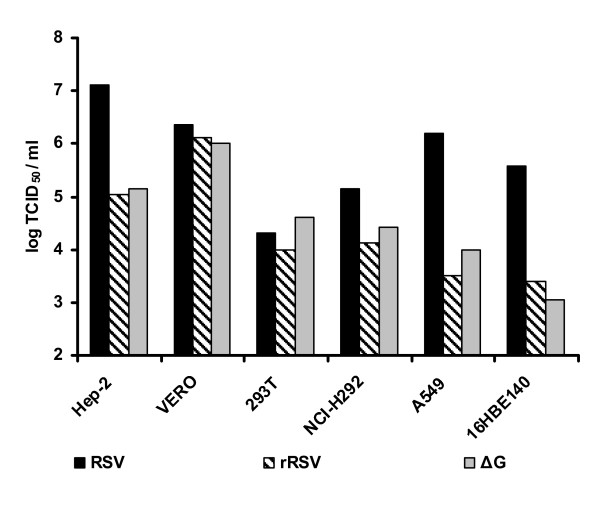
**RSV replication in cell lines**. Growth of RSV was tested in human lung mucoepidermoid carcinoma cells NCI-H292, human bronchial epithelial cell line 16HBE140, human lung epithelial carcinoma cells A549, human kidney epithelial cells 293T, human epithelial Hep-2 cells and monkey kidney Vero cells. Cells were infected with virus with an MOI of 0.1, harvested after 72 hr and virus CID_50 _titers were determined in Vero cells.

We next ascertained the growth kinetics of the recombinant viruses on different epithelial and kidney cell lines including lung mucoepidermoid carcinoma cells (NCI-H292), human bronchial epithelial cells (16HBE140), human lung epithelial carcinoma cells (A549), and human kidney epithelial cells (293T). To this aim, the cell lines were infected with an MOI of 0.1 and the viral titers were determined at 72 h post infection (Figure 3). In all cell types examined rRSV and ΔG showed similar growth characteristics. Wild type virus reached titers of 10^6 ^to 10^7 ^in the A549 and Hep-2 cells, whereas the recombinant viruses produced approximately 100-fold lower titers. A similar picture was found on the NCI-H292 cells, although wild type virus only reached moderate titers of 10^5.2 ^and the recombinant viruses had 10-fold lower titers. All (r)RSV strains showed similar poor growth on the 293T cells with maximum titers of 10^4.3^.

### Virus replication in cotton rats

Wild type and recombinant RSV-X strains were compared with the extensively well characterized subtype A strain RSV-A2 for their ability to replicate in the cotton rat lung. Animals were infected intranasally (i.n.) and were sacrificed after three, five or seven days post inoculation. Lung tissues and nasal lavages were collected for virus titration in Vero cells. High amounts of virus were found for both RSV-X and RSV-A2 in the lungs and nasal washes on day three and five, reaching titers of 10^5^; whereas low levels of virus could be isolated from the nose until day seven (Table 1). Replication of rRSV was 100-fold lower compared to wt RSV-X and reached maximum titers of approximately 10^2.8 ^in the lungs of half or three quart of the immunized animals, respectively at day three and five. At these days, most of the rRSV infected animals had low levels of virus in the nose. Replication of the ΔG mutant in the lungs and nose was below the limit of detection at any of the time points. Thus, although we did not observe a difference in replication kinetics between rRSV and ΔG *in vitro*, the absence of the G protein resulted in an attenuated phenotype of the latter virus in the cotton rat.

**Table 1:** Replication of (recombinant) RSV in the upper and lower respiratory tract of cotton rats.

Virus^a^	Day of harvest^b^	Lungs		Nose	
		
		% positive animals^c^	Mean titer ± SD (log_10_TCID_50_/g)^d^	% positive animals^c^	Mean titer ± SD (log_10_TCID_50_)^d^
RSV-A2	3	100	4.9 ± 0.3	100	3.0 ± 0.7
RSV-X	3	100	4.8 ± 0.3	100	3.7 ± 0.7
rRSV	3	50	2.8 ± 0.2	75	1.7 ± 0.3
ΔG	3	0	<2.1	0	<1.6
					
RSV-A2	5	100	5.1 ± 0.4	100	4.7 ± 0.2
RSV-X	5	100	4.7 ± 0.3	100	3.6 ± 0.5
rRSV	5	75	2.5 ± 0.2	100	2.2 ± 0.3
ΔG	5	0	<2.1	0	<1.6
					
RSV-A2	7	25	2.7	100	2.5 ± 0.8
RSV-X	7	0	<2.1	100	2.8 ± 0.9
rRSV	7	0	<2.1	ND	
ΔG	7	0	<2.1	ND	

^a ^Cotton rats were infected with 10^5 ^TCID_50 _(100 μl) of the indicated virus at day 0.

^b ^At the indicated days lungs and nasal washes were harvested and virus titers were determined.

^c ^Percentage of animals with detectable virus.

^d ^The lower limit of detection for virus in the lungs and nose was respectively 2.1 log_10_TCID_50 _g^-1 ^and 1.6 log_10_TCID_50_. Groups consisted of 4 animals. ND: not determined.

Pulmonary inflammation was assessed using a scoring scale system comparable to described by Prince [16]. Infection with either RSV-X or RSV-A2 induced a mild to moderate inflammation around peribronchi(oli), and in alveoli and bronchial mucous epithelium was moderately hypertrophied with a peak inflammation on day 5. Perivasculitis was sporadically seen and only in a minimal way. Infection pathology with rRSV was milder than wild type RSV-X. The ΔG mutant did not show any detectable inflammatory damage, which is in accordance with the inability to demonstrate replication of this virus in the lungs (data not shown). The wild type and viruses RSV-A2 or RSV-X, but not the recombinant viruses rRSV and ΔG, induced a minimal to slight modest infiltration of eosinophils in the epithelium of the bronchus.

### Protective efficacy in cotton rats

**Figure 4:**
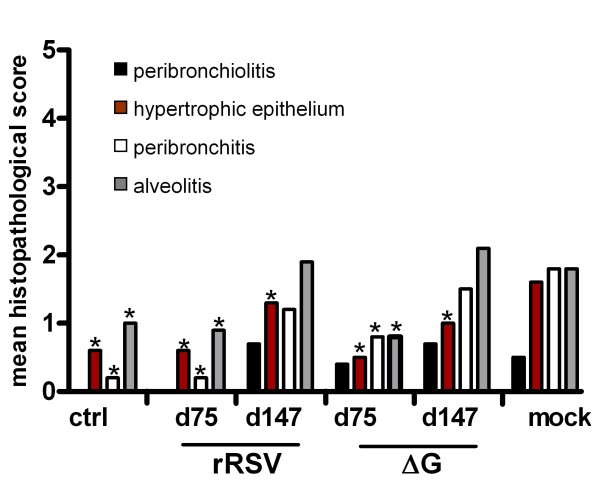
**Long term protection against RSV challenge lung histopathology in cotton rats**. Cotton rats were immunized i.n. at day 0 with 10^5 ^TCID_50 _rRSV or ΔG. Challenge was performed at day 70 and 142 with 10^6 ^TCID_50 _RSV-X (i.n) and the animals were sacrificed 5 days later, at day 75 and 147, respectively. Groups consisted of 6 animals. Mean histopathological scores of following histopathological parameters: peribronchiolitis (black bars), hypertrophied mucous cells (brown bars), peribronchitis (white bars) and alveolitis (gray bars). Ctrl: control animals; mock: mock infected, challenged animals. *: statistically significant different (P < 0.05) compared to mock infected group based on the Wilxocon test.

The recombinant viruses were subsequently examined for long term protective capacity against a pulmonary RSV challenge. To this aim, cotton rats were immunized with a single dose on day 0 and challenged on day 70 or 142. Five days after challenge infection, on day 75 or 147, respectively, the animals were sacrificed. A single immunization with either rRSV or ΔG induced sufficient immunity to protect the animals completely from challenge virus replication in the lungs up to 147 days after immunization (Table 2). rRSV immunization could protect the majority of animals against challenge virus replication in the nose, whereas ΔG could not. Both recombinant RSV-X viruses induced high serum RSV antibody titers at day 70, which dropped moderately at day 142. Furthermore, protection from pulmonary inflammation could be demonstrated until day 75 for both rRSV and ΔG (Figure 4). At this time, protection was significant for all histopathological parameters. Neither rRSV nor ΔG could fully protect against challenge histopathology at day 147.

**Table 2:** Long term protection after a single dose immunization in cotton rats

Immunization^a^	Challenge^b^		Mean virus titers post challenge ± SD^c^				Neutralizing antibodies at day of challenge (log_2_)^d^
			Lungs		Nose		
				
Vaccine	Day	Virus	% positive animals	(log_10_TCID_50_/g)	% positive animals	(log_10_TCID_50_)	
							
rRSV	70	RSV	0%	<2.1	17%	2.7	6.2
	142	RSV	0%	<2.1	33%	3.1 ± 0.5	4.9
							
ΔG	70	RSV	0%	<2.1	83%	3.7 ± 0.4	5.3
	142	RSV	0%	<2.1	83%	3.2 ± 0.7	4.2
							
mock	142	RSV	100%	5.0 ± 0.4	100%	3.8 ± 0.8	<3.3
mock	142	mock	0%	<2.1	0%	<1.6	<3.3

^a ^Cotton rats were infected with 10^5 ^TCID_50 _of the indicated virus at day 0. Groups consisted of 6 animals.

^b ^At the indicated days cotton rats were challenge i.n. with 10^6 ^TCID_50 _of RSV-X, and sacrificed 5 days post challenge.

^c ^Lungs and nasal washes were harvested and virus titers were determined. The lower limit of detection for virus in the lungs was 2.1 log_10_TCID_50 _g^-1 ^and in the nose 1.6 log_10_TCID_50_.

^d ^At the indicated days sera were collected and the neutralizing antibody titer against RSV-X was determined. The pre-infection serum titers were <3.3 (reciprocal log_2_) for all animals in the study.

## Discussion

This report describes that a recombinant RSV lacking the G protein (ΔG) is able to induce long-lasting protection against RSV challenge infection in cotton rats. A clinical isolate from 1998 from the Netherlands was used as source material for construction of the recombinant RSV. Compared to the parental recombinant rRSV, this ΔG virus was highly attenuated since replication of this virus in the cotton rat lungs could not be detected. Although attenuated, this virus could induce long-term protection against both wild type RSV challenge infection and the occurrence of infection-induced lung pathology.

This is the first description of a recombinant human RSV based on a clinical isolate. So far, the described recombinant RSVs have been based on the laboratory adapted A2 strain. These have been extensively studied, both *in vitro *and *in vivo *[8]. RSV-A2 was originally isolated in the 1960-s, while RSV-X is a clinical isolate from 1998. Although the growth characteristics of wild type RSV-X and its derived recombinant viruses were comparable in kidney epithelial cell lines like Vero and 293T, the recombinant viruses were attenuated in human epithelial cell lines like Hep-2, A549 and 16HBE140. Attenuation of recombinant RSV on Hep-2 cells has been reported in other studies [14,17] and is postulated to be a general feature of clonal RSV from one genetic sequence. Alternatively, to incorporate restriction endonuclease sites for cloning purposes, the intergenic regions of the RSV genome of the RSV-X clone differ 20 nucleotides from the wild type sequence. This might influence virus transcriptional regulation and explain the attenuated phenotype of the recombinant viruses observed in human epithelial cells [18].

A high and comparable level of virus titers in cotton rat lungs and nasal washes was observed for both RSV-X and RSV-A2, with peak titers occurring on day five after infection, similar as described previously [16]. In addition, both virus strains induced similar infection pathology in the lungs. In contrast, replication of recombinant RSV-X was 100-fold attenuated compared to wt RSV-X. Deletion of the G gene from recombinant RSV-X lacking the G protein abrogated its detectable replicative capacity *in vivo*. Thus, although we did not observe a difference between rRSV and ΔG *in vitro*, the absence of the G protein clearly attenuated the ΔG virus in the cotton rat lungs and nose.

Single immunization of cotton rats with the attenuated rRSV or ΔG viruses conferred long-term protection (147 days) against challenge RSV replication in the lungs and induced high titers of RSV serum neutralizing antibodies, although these antibodies wane in time. Although no virus could be detected in the lungs after immunization, low levels of virus could be detected in the nasal washes, especially with the ΔG virus. Both ΔG and rRSV conferred protection against pulmonary inflammation which lasted at least until day 75. At this day, there was no difference in lung inflammation between rRSV and ΔG immunized or uninfected animals, confirming that immunization with live attenuated RSV is not associated with RSV enhanced disease as occurs with FI-RSV vaccines. Protection against infection pathology waned sooner than protection against challenge virus replication. Mild inflammatory responses in the lungs were detected in animals immune to RSV, even in the absence of detectable virus replication. The detection levels of RSV lung titers might not have been sensitive enough to detect these low levels of RSV replication, since low levels of virus could be detected in the nasal washes.

The observed level of attenuation of the ΔG virus is consistent with previously reported data on cp52, a cold passaged RSV mutant lacking functional G and SH. This mutant was highly attenuated in cotton rats, attenuated and immunogenic in chimpanzees, but was found to be overattenuated for RSV-seronegative infants and children [19]. However, this mutant possesses in addition to the deletion of functional G and SH, an aberrant SH-G intergenic region and mutations in the F and L gene. A number of recombinant RSV-A2 have been made that lack either the complete G gene or express truncated, secreted or membrane forms of G, but only a recombinant RSV-A2 with the membrane form of G has been tested for its protective efficacy in BALB/c mice [20]; but this mutant was considered to be over-attenuated. However, the attenuated nature of this mutant is not convincing; other papers describe that such a recombinant RSV-A2 showed enhanced virulence compared to wild type RSV-A2 [21,22].

A recombinant bRSV lacking the G gene induced bRSV neutralizing antibodies after intranasal immunization of calves. Although it was not clear whether this mutant was able to replicate in the lungs, calves were protected against a subsequent bRSV challenge infection after mucosal administration [13]. Human metapneumovirus (HMPV) belongs to the same subfamily of the Pneumovirinae as RSV, recombinant viruses lacking the G gene are replication competent in the upper and lower respiratory tract of hamsters and were attenuated compared to wt HMPV. Intranasal immunization of HMPV lacking the G gene conferred protection against challenge virus replication in the lungs but not in the nasal turbinates [23]. These results are in agreement with our results. More importantly, we have shown that this highly attenuated ΔG confers long-term protection against subsequent challenge.

Deletion and/or functional inactivation of the gene coding for the G protein prevents a number of problems and complications associated with potential RSV vaccine candidates. One purpose is vaccine safety: RSV without G is highly attenuated in its host because it will not be able to infect host cells efficiently [13,19], is not likely to revert to a less attenuated phenotype and does not show enhanced disease. Moreover, a substantial role for the G protein has been suggested in the induction of undesired immunological responses, including enhanced immune pathology [24] and possible skewing of the immune system towards an allergy (and asthma) prone state under certain genetic predispositions [25]. In contrast, several recent studies have shown that G is not implicated in vaccine enhanced disease induced by immunization with formalin-inactivated RSV [26]. Nevertheless, candidate RSV vaccines will have to prove they will not induce enhanced disease in vaccinees.

## Conclusions

Our data represent the first characterization in a relevant animal model of a recombinant RSV lacking the G gene that can be considered safe because of it is high attenuation profile *in vivo *(no detectable virus replication in the cotton rat lungs and nasal washes) and its lack of induction of enhanced disease in the cotton rat model. Moreover, it has the capacity to induce long lasting protection against challenge virus replication. This study demonstrates that the attenuated recombinant RSV and the highly attenuated recombinant RSV virus lacking the G gene confer long-term protection against challenge virus replication and inflammation after a single immunization. The RSV lacking the G gene is not likely to revert to a less-attenuated phenotype, we plan to evaluate this mutant further to investigate its potential as a vaccine candidate against RSV infection.

## Methods

### Cells and viruses

Monkey kidney Vero cells (CCL-8, American Type Culture Collection (ATCC)) were cultured (37°C, 5% CO_2_) in M199 medium (Invitrogen) supplemented with heat-inactivated 5% fetal bovine serum (FBS, Hyclone) and PSG (100 units of penicillin, 10 μg of streptomycin and 292 μg of L-glutamine/ml, Invitrogen). Hep-2 (CCL-23, ATCC) human lung epithelial carcinoma cells A549 (CCL-185, ATCC) and human kidney epithelial cells 293T (CRL-11268, ATCC) lung mucoepidermoid carcinoma cells NCI-H292 (CRL-1848, ATCC), human bronchial epithelial cell line 16HBE14o [27] were cultured in DMEM medium (Invitrogen) with 10% FBS and PSG. RSV infected cells were grown at 37°C in DMEM medium supplemented with 1% FCS and PSG. The following RSV strains were used: A2 (ATCC, VR1302) and clinical isolate 98-25147-X (Leiden University Medical Centre, The Netherlands). The latter virus was isolated in 1998 and propagated for 9 passages on Hep-2 cells (CCL-23, ATCC). This stock was designated as RSV-X [Genbank FJ948820] and was used for generation of cDNA. RSV-X virus was determined as a subtype A antigenic isolate and genotyped as a GA2 virus. Modified vaccinia virus Ankara expressing T7 RNA polymerase (MVA-T7) was kindly provided by G. Sutter (Paul-Ehrlich Institute, Langen, Germany).

### Construction of cDNA encoding RSV-X and RSV-X lacking the G gene

The antigenomic cDNA spanning the entire RSV-X strain genome was assembled into a single molecule by sequential ligation of RSV X cDNA fragments (Figure 1). This was facilitated by sequential cloning six cDNA fragments of the RSV genome into one expression vector to create a complete full length cDNA. Each fragment was sequenced completely using an ABI 310 DNA sequencer (Applied Biosystems). Five restriction sites (*SexA I, Xma I, Bswi I, BssH II and Mlu I*) were artificially introduced in the intergenic regions during the cloning procedure to help in cloning as well as to serve as markers to confirm the identity of the recovered recombinant virus. cDNA fragments were generated by reverse transcriptase (RT) PCR performed with Thermoscript reverse transcriptase (Invitrogen) and High fidelity platinum *Taq *polymerase (Invitrogen) using RSV-specific primers based on the sequence of RSV-A2 (primer sequences are available upon request). The first cDNA fragment had a T7 RNA polymerase promoter sequence located immediately proceeding nucleotide (nt) 1 and encompassed the leader sequence, NS1, NS2 and had an engineered *SexA I *site at nt position 1246. The second fragment encompassed N (nt 1246 to 2467) and was flanked by restriction sites *SexA I *and *Xma I*. The third fragment encompassed P, M, SH (nt 2467 to 4800) and was flanked by restriction sites *Xma I *and *Bswi I*. The fourth fragment encompassed G (nt 4800 to 5730) and was flanked by restriction sites *Bswi I *and *BssH II*. The fifth fragment encompassed F, M2.1 M2.2 (nt 5730 to 8587) and was flanked by restriction sites *BssH II and Mlu I*. The sixth fragment encompassed L, the trailer sequence (nt 8587 to 15213), the hepatitis delta virus ribozyme followed by a terminator of the T7 polymerase and was flanked by restriction sites *Mlu I *and *Kpn I*. In addition to the construction of a full length recombinant RSV-X cDNA clone (pRSV-X), a recombinant RSV-X cDNA clone lacking the G gene was recovered from the full length clone by excision of the *BssH II *and *BsiW I *fragment, followed by religation (pRSV-XΔG). The full length cDNA clones were verified by sequencing completely.

### Recovery of recombinant viruses

Recombinant RSVs were recovered from cDNA largely as described before [17,28]. MVA-T7 infected Hep-2 cells were transfected with the antigenic plasmid (pRSV-XΔG or pRSV-X) and a set of four helper plasmids expressing the RSV N, P and M2.1 proteins (designated pcDNA6-A2-N, pcDNA3-A2-P, pcDNA6-A2-L, and pcDNA6-A2-M2.1 respectively). These helper plasmids expressed RSV genes of RSV strain A2. The amounts of the plasmids added was as follows: 1.6 μg pRSV-XΔG or pRSV-X, 1.6 μg pcDNA6-A2-N, 1.2 μg pcDNA3-A2-P, 0.4 μg pcDNA6-A2-L, 0.8 μg pcDNA6-A2-M2.1. After 3 - 4 hrs of incubation at 32°C, 500 μl of Optimem (Invitrogen) with 2% FCS was added and the cells were incubated at 32°C for 3 days. Cells were then scraped and the mixture of scraped cells and medium containing the rescued virus was used to infect fresh cultures of Vero cells grown in DMEM + 1% FCS + PSG. The latter procedure was repeated for 4 - 5 times to amplify rescued virus. The viruses were purified and concentrated by PEG precipitation of culture supernatants and stored as stocks at -80°C.

### Characterization of recombinant viruses

Expression of the RSV proteins in infected Vero monolayers was confirmed by immunostaining with an anti-RSV goat polyclonal antibody (Biodesign), a monoclonal antibody against RSV-G (Mab 131-2G, Chemicon or Mab L9), or a monoclonal antibody against RSV-F (130-8F, Chemicon). Vero cells were infected at an m.o.i. of 0.1 TCID_50_/ml. At 72 hr post infection cell monolayers were harvested and approximately 5 × 10^4 ^cell equivalent was subjected to electrophoresis on a 12% SDS-polyacrylamide gel (Pierce), transferred to optitran BA83 nitrocellulose membrane (Whatman) and subjected to Western blotting using a mixture of Mab L9, Mab 130-8F and polyclonal RSV antibody. In addition, the identities of the recombinant viruses were verified by sequencing viral RNA of the rescued viruses. Growth analysis of recombinant viruses was determined by infecting 50 - 60% subconfluent Vero or Hep-2 cells with at MOI of 0.1. The infected monolayers were incubated at 37°C. At 0, 24, 48, 72, 96, and 120 hr post infection, cells and media were harvested and stored at -80°C.

### Viral titration

Virus titers were determined by 50% tissue culture infective dose (TCID_50_). TCID_50 _assays were first visually inspected for cytopathic effect after 7 days incubation at 37°C. The supernatants were subsequently analyzed by antigen capture ELISA using goat polyclonal antibody against RSV. To determine RSV titers in the lungs of cotton rats, the right lungs were removed, weighed, homogenized in stabilizing buffer, and stored at -80°C. RSV lung titers were determined on Vero cells and expressed in log_10_TCID_50 _per gram lung; the lowest limit of detection in cotton rat lungs was 2.1 log_10 _TCID_50_.g^-1^. To determine RSV titers in the nose, nasal washes were obtained by flushing the upper trachea with 2 ml PBS with 7.5% sucrose.

### Virus neutralizing assay

Two-fold serial dilutions starting at 1:10 of cotton rat serum were prepared in virus diluent (DMEM supplemented with 1% FCS and PSG). Each serum was mixed with an equal volume of virus (50-100 plaques/well) and incubated for 1 hr at 37°C. Vero monolayers, prepared in 96-well plates, were infected with 50 μl/well (in triplicate) of the serum/virus mixture. After centrifugation for 1 h at 700 × *g *and incubation of 1 hr at 37°C, supernatant was removed and cells were overlaid with 1.0% methyl cellulose prepared in DMEM supplemented with 1% FCS and PSG. After 2 days at 37°C, the overlay was removed and the cells were fixed with 80% acetone and stained with polycolonal anti-RSV HRP. Plaques were counted and plaque reduction was calculated by regression analysis to provide a 60% plaque reduction titer.

### Immunizations and RSV challenge

Cotton rats *(Sigmodon hispidus) *were originally obtained from Charles River Laboratories (Netherlands) and used for establishment of an in-house specific pathogen free breed. Unless otherwise specified, young (4 to 8 weeks) adult cotton rats were intranasally (i.n.) immunized at day 0 with 100 μl 10^5 ^TCID_50 _RSV-X, rRSV, or ΔG virus preparations. Each group consisted of 4 - 6 animals. At indicated time points the animals were challenged i.n. with 100 μl 10^6 ^TCID_50 _RSV-X. Mock immunized animals received PEG precipitated supernatants of mock infected Vero cells. Control animals were mock immunized and mock challenged. All infections were administered under anesthesia.

### Histopathology

The left lung was inflated intratracheally with 10% neutral buffered formalin and fixed for at least 24 hours. Lung tissue was longitudinal embedded in paraplast, haematoxilin and eosin (HE) stained sections (5 μm), in which all air passages from bronchi to terminal bronchioles and alveolar ducts were present, were judged light microscopically. A whole section of a lung was considered in the evaluation of the following histopathological parameters: hypertrophy of bronchial (mucous) epithelium, the presence of subepithelial inflammatory cells around bronchi (peribronchitis), bronchioles (peribronchiolitis) and blood vessels (perivasculitis) and in the alveoli (alveolitis), comparable to Prince *et al. *[16]. In addition the presence of intra-epithelial eosinophils was scored in the bronchus. These histopathological parameters were each semi quantitatively scored in a blinded manner by a pathologist as 0 = absent, 1 = minimal, 2 = slight, 3 = moderate, 4 = strong, and 5 = severe respectively. In this score, the frequency as well as the severity of the lesions was incorporated. For each group the mean score of each histopathological parameter was calculated. Since the scoring was non-linear, the histological data were analyzed by using the nonparametric Wilxocon test. Results for which the P value was <0.05 was considered to be significant.

## Competing interests

This work is part of collaboration with Nobilon International BV, which funded part of the study.

## Authors' contributions

MW participated in design and interpretation of the experiments, performed the research, and wrote the manuscript. JB carried out the virological experiments. MVB performed cloning experiments. YVR performed virus neutralization assays. PJMR carried out the pathological analysis and helped draft the manuscript. WL conceived the study and was involved in drafting the manuscript. All authors approved the final version of the manuscript.
